# Seasonality of active tuberculosis notification from 2005 to 2014 in Xinjiang, China

**DOI:** 10.1371/journal.pone.0180226

**Published:** 2017-07-05

**Authors:** Atikaimu Wubuli, Yuehua Li, Feng Xue, Xuemei Yao, Halmurat Upur, Qimanguli Wushouer

**Affiliations:** 1 Department of Epidemiology and Biostatistics, School of Public Health, Xinjiang Medical University, Urumqi, Xinjiang, China; 2 Center for Tuberculosis Control and Prevention, Xinjiang Uygur Autonomous Region Center for Disease Control and Prevention, Urumqi, Xinjiang, China; 3 Department of Traditional Uygur Medicine, Xinjiang Medical University, Urumqi, Xinjiang, China; 4 Department of Respiratory Medicine, The First Teaching Hospital of Xinjiang Medical University, Urumqi, Xinjiang, China; Hebrew University, ISRAEL

## Abstract

**Objectives:**

Xinjiang is one of the highest TB-burdened provinces of China. A time-series analysis was conducted to evaluate the trend, seasonality of active TB in Xinjiang, and explore the underlying mechanism of TB seasonality by comparing the seasonal variations of different subgroups.

**Methods:**

Monthly active TB cases from 2005 to 2014 in Xinjiang were analyzed by the X-12-ARIMA seasonal adjustment program. Seasonal amplitude (SA) was calculated and compared within the subgroups.

**Results:**

A total of 277,300 confirmed active TB cases were notified from 2005 to 2014 in Xinjiang, China, with a monthly average of 2311±577. The seasonality of active TB notification was peaked in March and troughed in October, with a decreasing SA trend. The annual 77.31% SA indicated an annual mean of additional TB cases diagnosed in March as compared to October. The 0–14-year-old group had significantly higher SA than 15–44-year-old group (*P*<0.05). Students had the highest SA, followed by herder and migrant workers (*P*<0.05). The pleural TB cases had significantly higher SA than the pulmonary cases (*P* <0.05). Significant associations were not observed between SA and sex, ethnic group, regions, the result of sputum smear microcopy, and treatment history (*P*>0.05).

**Conclusion:**

TB notification in Xinjiang shows an apparent seasonal variation with a peak in March and trough in October. For the underlying mechanism of TB seasonality, our results hypothesize that winter indoor crowding increases the risk of TB transmission, and seasonality was mainly influenced by the recent exogenous infection rather than the endogenous reactivation.

## Introduction

Tuberculosis (TB) remains one of the most lethal communicable diseases worldwide, which is estimated to 9 million as advanced TB cases and 1.5 million deaths in 2013 [1]. It has been estimated that although one-third of the global population has been infected by *Mycobacterium tuberculosis*, only a minority, probably about 10% develop active tuberculosis [2]. The occurrence of active tuberculosis includes the development of the disease due to recent exogenous infection/reinfection and endogenous reactivation of long-term latent infection. The proportion of recent exogenous infection cases was higher in developing countries while the endogenous reactivation was the primary cause of TB in developed countries [3–4].

Several studies have identified the seasonality of TB, which indicated the peak month of TB notification in spring and/or early summer and trough month in late autumn or winter [5–7]. The spring peak of TB notification suggested the peak of infection and the onset of disease in winter after considering the latency of the disease, diagnosis, and treatment delay. Some explanations were proposed for the seasonality of TB notification. Firstly, vitamin D deficiency in winter decreases the immune competence, thereby increasing the endogenous reactivation of latent infection, as well as, the risk of disease development of recent exogenous infection. Secondly, winter indoor crowding increased the transmission of TB, suggesting the seasonal occurrence of increase in the disease by recent exogenous infection rather than the relapse of latent disease. Thirdly, the co-infection of other seasonal respiratory viral pathogens such as chickenpox and measles also increased the risk of TB. Lastly, the reporting bias means that the access to health care is maybe more difficult at the certain duration of the year. Understanding the determinants of the seasonality of TB further elucidate the pathogenesis of TB influencing factors and suggest the preventive, therapeutic measures.

Several time-series analysis methods such as Fourier analysis, cosinor analysis, sinusoidal harmonic model, spectral analysis, seasonal autoregressive integrated moving average model (SARIMA), TRAMO-SEATS, X-12-ARIMA, and others were used to analyze the seasonality. However, X-12-ARIMA was considered as the unsurpassed seasonal adjustment model [8].

China ranks second in high TB-burdened countries, and Xinjiang is one of the highest TB-burdened provinces of China. It is also the largest political subdivision of China (accounting for more than one-sixth of the total territory and a quarter of the boundary length of the country) and is the home to 47 different ethnic groups. To the best of our knowledge, a relevant study about TB seasonality in Xinjiang is yet lacking. The present study aimed to evaluate the trend, seasonality of active TB in Xinjiang, and explore the underlying mechanism of the TB seasonality by comparing the seasonal variations of different subgroups.

## Methods

### 2.1 Data sources

The data of confirmed active TB cases from January 2005 to December 2014 was obtained from National Infectious Diseases Reporting System (NIDRS) database and the Chinese Center for Disease Control and Prevention. The month was used as the unit of this time series study.

Daily records were aggregated into the month, and the series of monthly cases created 120 data points. The monthly notifications of TB cases were also collected by different subgroups, including age group, sex, ethnic group, occupation, region, disease site, the result of sputum smear microscopy, and treatment history.

Ethics: The data from TB surveillance system were aggregated as secondary data without any personal information, and thus, informed consent was not required. The study was approved by the Ethics Committee of The First Teaching Hospital of Xinjiang Medical University.

### 2.2 Statistical methods and software

The monthly notification of TB cases was analyzed by SAS version 9.2 using X-12-ARIMA program of time-series decomposition method developed by the US Census Bureau in 1998 [9]. X-12-ARIMA is currently the most widely used model and is also considered as the best seasonal adjustment method. The original time series are usually decomposed into (1) the trend component that reflects the long-term progression of the series, (2) the seasonal component that reflects seasonal variation, and (3) the irregular component (or "noise") that describes random, irregular influences.

A decomposition of monthly notification was conducted for each subgroup of interest. The mean peak month, trough month and annual seasonal amplitude (SA) with 95% confidence intervals (CIs) for each subgroup were calculated if identifiable seasonality was assessed by X-12-ARIMA. The annual SA was calculated from isolated seasonal factor and defined as the fraction with the numerator representing the peak-to-trough difference between the months with the highest and the lowest case counts and the denominator as the mean case counts for that year. CIs were calculated using the Wald method based on the variance of the mean of 10 annual amplitude measurements. The Student’s t-test for two independent samples was used to compare the SAs of two subgroups. The Bonferroni method for one-way analysis of variance (ANOVA) was used to compare all pairwise SAs of three or more subgroups in equal variance and normal distribution, whereas the Kruskal-Wallis test was used for an unequal variance or skewed distribution. *P*-value< 0.05 was considered statistically significant.

## Results

To A total of 277,300 confirmed active TB cases were notified from 2005 to 2014 in Xinjiang, China. The notification of active TB cases in different subgroups was presented in Table 1. The sex ratio of the TB cases was 1.12:1 and most of them were 45–64 years old (33.6%), Uygur (66.6%), peasant (74.2%), and from Southern Xinjiang (72.0%). The vast majority of the notified active cases were pulmonary TB (99.2%) and the ratio of sputum smear-positive and -negative cases was 0.83, whereas the relapse cases accounted for 20.6% of sputum smear-positive TB cases.

**Table 1:** Distribution of active TB notification in Xinjiang from 2005 to 2014 (Unit: thousand).

Group		2005	2006	2007	2008	2009	2010	2011	2012	2013	2014	Total
All active TB cases		26.71	30.61	29.55	28.30	23.90	25.47	23.13	28.17	30.44	31.02	277.30
Sex	Male	14.09	16.22	15.61	15.23	12.66	13.45	12.22	15.01	15.89	16.16	146.55
	Female	12.62	14.39	13.94	13.07	11.24	12.02	10.91	13.16	14.55	14.86	130.75
Age	0–14	0.49	0.45	0.36	0.31	0.31	0.30	0.25	0.23	0.19	0.24	3.13
	15–24	4.07	4.38	3.85	3.79	3.56	3.57	3.36	3.25	3.22	3.10	36.15
	25–44	7.51	8.10	7.38	7.24	6.34	6.79	5.98	6.96	6.94	7.06	70.28
	45–64	8.87	9.95	9.78	9.14	7.63	8.27	7.56	9.89	10.80	11.29	93.16
	65+	5.78	7.72	8.18	7.83	6.06	6.55	5.99	7.85	9.29	9.34	74.59
Ethnicity	Han	6.36	6.96	6.74	6.49	5.52	5.89	5.44	6.35	6.86	7.10	63.69
	Uygur	17.64	20.57	19.88	18.78	15.74	16.83	15.12	18.88	20.56	20.82	184.82
	Kazak	1.28	1.46	1.42	1.50	1.31	1.33	1.25	1.42	1.39	1.48	13.84
	Others	1.43	1.62	1.52	1.53	1.33	1.41	1.32	1.52	1.64	1.63	14.94
Occupation	Peasant	20.01	22.84	22.09	20.88	17.29	18.35	16.46	20.81	23.26	23.78	205.75
	Herder	0.76	0.84	0.72	0.73	0.68	0.68	0.55	0.71	0.51	0.45	6.62
	Migrant worker	0.31	0.33	0.30	0.28	0.31	0.36	0.36	0.42	0.29	0.21	3.16
	Unemployed	1.38	1.59	1.64	1.61	1.40	1.53	1.74	1.96	2.30	2.56	17.72
	Students	0.88	1.07	0.92	1.01	1.03	1.05	0.95	0.85	0.87	0.97	9.60
	Others	3.36	3.94	3.88	3.79	3.19	3.50	3.07	3.42	3.21	3.05	34.45
Regions	Northern Xinjiang	6.31	6.99	6.81	7.11	6.37	6.89	6.37	6.93	7.33	7.69	68.8
	Southern Xinjiang	19.55	22.6	21.79	20.28	16.67	17.78	16.01	20.35	22.18	22.44	199.65
	Eastern Xinjiang	0.85	1.02	0.95	0.91	0.86	0.8	0.75	0.89	0.93	0.89	8.85
Disease Site	Pulmonary TB	26.36	30.23	29.21	27.99	23.75	25.21	23.02	28.07	30.36	30.85	275.03
	Pleural TB	0.34	0.37	0.30	0.24	0.14	0.22	0.11	0.10	0.08	0.17	2.06
Sputum smear microscopy	Positive TB	17.35	16.90	15.48	14.18	12.14	11.42	9.40	11.00	8.46	7.93	124.25
	Negative TB	8.90	13.22	13.59	13.81	11.61	13.74	13.56	17.06	21.88	22.91	150.29
Sputum smear positive	New case	14.32	13.96	12.49	11.25	9.46	9.01	7.29	8.55	6.42	5.89	98.63
	Relapse case	3.03	2.94	2.99	2.92	2.69	2.41	2.11	2.45	2.05	2.04	25.62

The average number of cases notified per month was 2311±577 (minimum = 991, maximum = 3941). The proportion of monthly reported TB cases from January to December was 8.2, 8.8, 10.7, 10.2, 9.9, 8.6, 7.7, 8.0, 7.3, 5.7, 7.9, and 7.0%, respectively. The monthly reported active TB cases from 2005 to 2014 were listed in Table 2.

**Table 2:** Monthly notification of active TB in Xinjiang from 2005 to 2014.

	2005		2006		2007		2008		2009		2010		2011		2012		2013		2014	
Month	N	%	N	%	n	%	n	%	N	%	n	%	n	%	n	%	n	%	N	%
Jan	1931	7.23	1863	6.09	3034	10.27	2809	9.93	1803	7.54	2459	9.66	2055	8.88	1234	4.38	2595	8.52	2814	9.07
Feb	1751	6.56	3101	10.13	2808	9.50	2550	9.01	2550	10.67	1935	7.6	1851	8.00	2613	9.27	2510	8.24	2736	8.82
Mar	2869	10.74	3465	11.32	3148	10.65	3246	11.47	2744	11.48	2433	9.55	2566	11.09	2634	9.35	3154	10.36	3338	10.76
Apr	2648	9.91	3288	10.74	3187	10.79	3066	10.83	2365	9.89	3485	13.68	2571	11.11	1873	6.65	2592	8.51	3161	10.19
May	2770	10.37	3045	9.95	2769	9.37	2688	9.5	2208	9.24	2504	9.83	2516	10.88	2757	9.79	3130	10.28	3084	9.94
Jun	2432	9.11	2824	9.23	2330	7.89	2167	7.66	2309	9.66	2550	10.01	2154	9.31	2066	7.33	2357	7.74	2708	8.73
Jul	1925	7.21	2461	8.04	2377	8.04	2527	8.93	1663	6.96	1978	7.77	1856	8.02	1950	6.92	2671	8.77	2056	6.63
Aug	2378	8.90	3067	10.02	2356	7.97	2092	7.39	1812	7.58	2266	8.9	2009	8.69	1788	6.35	2189	7.19	2109	6.80
Sep	2212	8.28	2424	7.92	2051	6.94	2020	7.14	1750	7.32	1190	4.67	991	4.28	2530	8.98	2680	8.80	2398	7.73
Oct	1479	5.54	1575	5.15	1631	5.52	1369	4.84	1292	5.41	1392	5.47	1421	6.14	1508	5.35	2143	7.04	2067	6.66
Nov	2329	8.72	2252	7.36	2149	7.27	2098	7.41	1556	6.51	1410	5.54	1354	5.85	3941	13.99	2609	8.57	2211	7.13
Dec	1986	7.44	1244	4.06	1709	5.78	1667	5.89	1849	7.74	1864	7.32	1787	7.73	3280	11.64	1813	5.96	2336	7.53
Total	26710	100	30609	100.00	29549	100.00	28299	100	23901	100	25466	100	23131	100.00	28174	100	30443	100.00	31018	100.00

**Fig 1:**
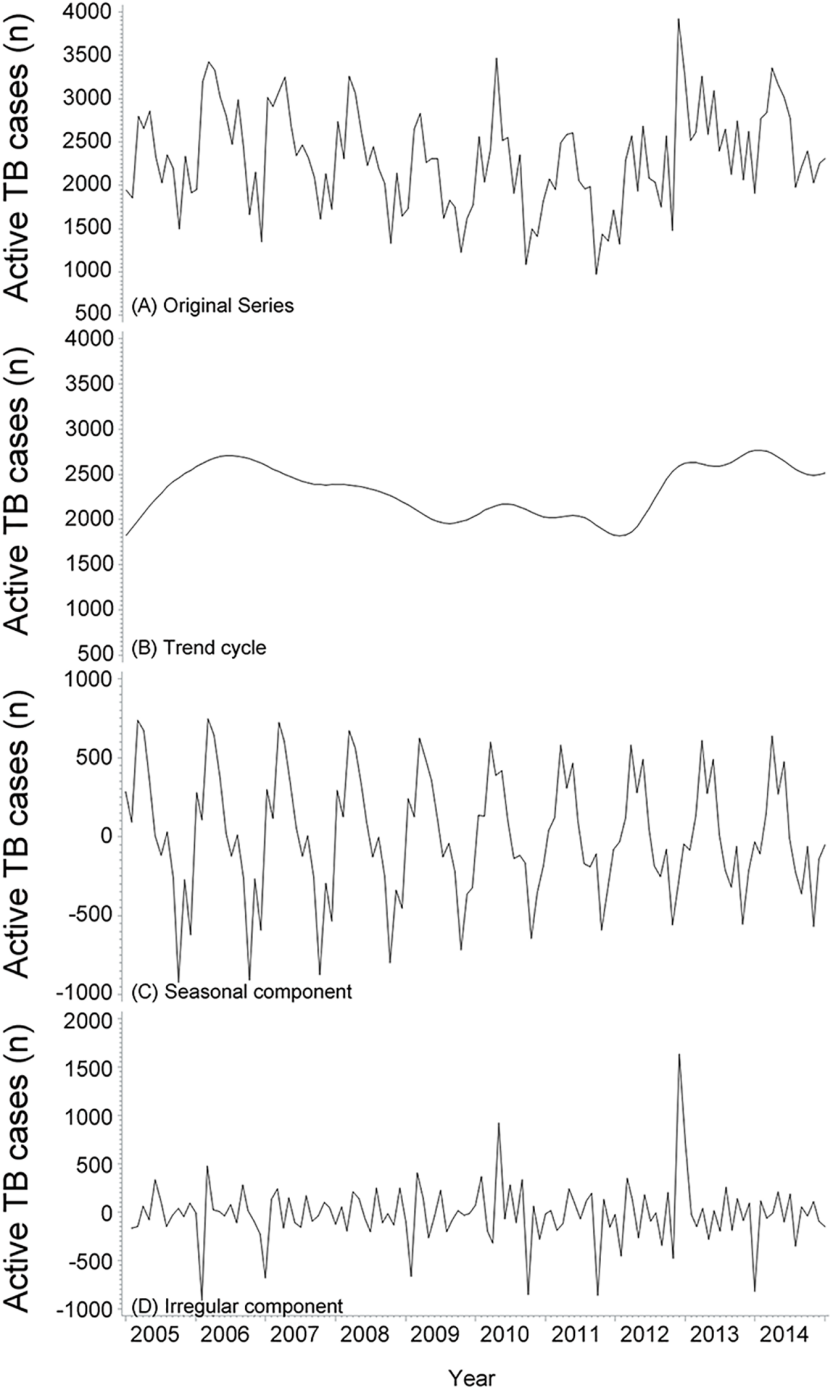
Original time series and X-12-ARIMA seasonal decomposition of monthly notification of active TB in Xinjiang from 2005 to 2014: original series (A) with trend (B), seasonal component (C), and irregular component (D).

Fig 1A demonstrated the original time series of monthly notification of active TB cases displaying consistent annual periodicity and seasonal fluctuation. The original time series were decomposed into trend cycle (Fig 1B), seasonal (Fig 1C), and irregular (Fig 1D) components by the X-12-ARIMA seasonal adjustment method. Fig 1B demonstrated that the isolated trend cycle curve smoothened after excluding the seasonal and irregular variations. The isolated trend cycle showed an increasing trend of active TB cases from 2005 to 2006, a decreasing trend from 2006 to 2011, followed by an upward trend from 2011 to 2014. Fig 1C showed an apparent seasonality of active TB notification in Xinjiang, with a peak in the month of March and trough in the month of October, and the SA had a decreasing trend from 2005 to 2014. Fig 1D represented an irregular component including random fluctuations, abnormal values, and other irregular factors. An abnormal peak of TB notification was observed in November 2012 and April 2010, indicating a possible outbreak. On the other hand, the abnormal trough months were found in January and December 2006, January 2009, September 2010, September 2011, and December 2013, likely attributable to the impact of the unadjusted holidays.

The analysis of the isolated seasonal component revealed that the annual SA for total active TB cases was 77.31% (61.63–92.98), suggesting an annual mean of 77.31% additional cases of TB diagnosed in peak month (March) as compared to the trough month (October). Table 3 displayed the peak and trough month, SA, and 95% CI of SA for the population of interest. Comparison of SA between different subgroups showed that the magnitude of seasonality was associated with age, occupation, and disease site. The 0–14-year-old group had significantly higher SA than 15–44-year-old group (*P*<0.05). Students showed the highest SA, followed by herder and migrant workers (*P*<0.05). Students had a late peak month of TB notification compared to the total cases. The pleural TB cases had significantly higher SA compared to the pulmonary TB cases (*P*<0.05). No significant associations were observed between SA and sex, ethnic group, regions, the result of sputum smear microscopy, and treatment history (*P*>0.05).

**Table 3:** Comparisons of seasonal amplitude of active TB notification among different subgroups in Xinjiang.

Group		Peak/ Trough Month	Mean seasonal amplitude (%)	SE (%)	95% CI (%)	*P* value
All active TB cases		March / October	77.31	6.93	61.63–92.98	
Sex	Male	March / October	75.00	7.08	58.98–91.02	> 0.05
	Female	March / October	80.71	6.82	65.30–96.13	
Age	0–14	March / January	109.56	8.07	91.30–127.82	< 0.05
	15–24	May / October	80.40	4.61	69.98–90.82	
	25–44	March / October	70.58	4.36	60.72–80.44	
	45–64	March / October	85.61	7.28	69.13–102.09	
	65+	March / October	88.30	10.29	65.02–111.58	
Ethnicity	Han	March / October	69.00	7.14	52.84–85.16	> 0.05
	Uygur	March / October	83.96	7.56	66.85–101.06	
	Kazak	April / October	75.40	8.82	55.45–95.35	
	Others	April / October	76.57	6.18	62.58–90.56	
Occupation	Peasant	March / October	86.37	8.35	67.48–105.25	< 0.05
	Herder	November / September	111.38	14.92	77.62–145.13	
	Migrant worker	April / February	102.00	4.82	91.09–112.90	
	Unemployed	March / October	81.00	3.96	72.05–89.95	
	Students	May / December	129.21	5.91	115.85–142.57	
	Others	April / October	60.10	4.26	50.48–69.73	
Regions	Northern Xinjiang	April / October	72.88	5.40	60.66–85.11	> 0.05
	Southern Xinjiang	March / October	84.83	7.80	67.19–102.47	
Disease Site	Pulmonary TB	March / October	77.46	6.98	61.68–93.24	< 0.05
	Pleural TB	May / October	178.31	30.40	109.53–247.08	
Sputum smear microscopy	Positive TB	March / October	75.86	10.65	51.78–99.94	> 0.05
	Negative TB	March / October	89.19	5.83	76.01–102.37	
Sputum smear positive	New case	March / October	74.27	12.27	46.51–102.03	> 0.05
	Relapse case	March / December	94.92	8.41	75.90–113.95	

## Discussions

The present study showed an apparent seasonal variation of TB notification in Xinjiang, China, with a peak in the month of March and trough in the month of October. The spring peak month was similar to the results from many other studies conducted in the northern hemisphere including those in India [10], Spain [11], Mongolia [12], China [13], the USA [14], Portugal [15], and the Netherlands [16]. The peak TB notification in South Africa [17] and Australia [18] was in October, which is the month of local spring in the southern hemisphere. On the other hand, the summer peak month was suggested in countries like United Kingdom [19] and Hong Kong [20] while others found double peak months in spring and summer such as Wuhan (China) [21], Japan [22], and Kuwait [23]. The autumn trough months of the present study were similar to those from the USA [14] and India [10]; however, it was earlier than the winter trough month of all the other regions in China [13]. The variable peak month of the active TB notification was correlated with several factors such as location, latitude, climate, customs, economy, and the TB burden of those countries.

There were several explanations for the spring peak of TB notification. The spring peak month indicated the greater risk of TB transmission in winter after considering the incubation period and diagnosis delay [24]. Our results support the hypothesis that winter indoor crowding increases the risk of TB transmission, and the TB seasonality was primarily influenced by the recent exogenous infection rather than endogenous reactivation in Xinjiang.

### 3.1 Winter indoor crowding hypothesis

The indoor activity of the population increases commonly in winter due to cold weather, and the overcrowding, poor ventilation, and high level of humidity provide a suitable environment for the survival of *M*. *tuberculosis*. The overcrowded transportation during the spring festival in China had also been postulated as a risk factor for TB transmission in winter [13]. Winter indoor and/or traffic crowding increases the number of TB cases due to recent exogenous infection/re-infection rather than endogenous reactivation [14]. The evidence supporting this hypothesis in the present study is as follows:

Firstly, the degree of seasonality was greater among children (0–14 years age group) who were more likely to succumb to recent infection of *M*. *tuberculosis* as compared to the elderly who may have been infected decades earlier. Similar results were also found in other studies [13–14]. Secondly, when comparing the SA of different occupational groups, students showed significantly higher SA and later peak month than others. The winter vacation of students was from mid-January to mid-February in China, after which, they returned to school in a relatively crowded environment such as dormitories and classroom. Moreover, the college students also returned to school concurrently, thereby causing an additional traffic peak, and the relatively crowded transportation such as trains and buses would increase the risk of exogenous TB infection. Thus, the peak month of TB notification was in May after considering the diagnosis delay and incubation period. Thirdly, the degree of seasonality was greater in high TB-burdened regions and high-risk population. Southern Xinjiang, which has consistently been the hotspot of TB incidence in Xinjiang, had greater SA of TB notification than northern and eastern Xinjiang. Uygur ethnic group, a high TB-risk population, had greater seasonality of TB than others; however, these differences failed to reach statistical significance. Additional infection sources occurred in high-TB-burdened regions and high-risk population that is likely to be affected by the increased risk of TB transmission in winter indoor crowding, thereby resulting in a large SA than others. Lastly, the SA had a decreasing trend from 2005 to 2014, which coincided with the downward trend of TB notification. Although there was an increasing trend of active TB notification since 2011, it was mainly caused by the increase of sputum smear-negative TB cases; the leading infection source sputum-smear positive TB cases were consistently decreasing since 2006 according to our previous study [25]. The decline of infection source would minimize the effect of increased TB transmission in winter indoor crowding, due to which, the SA also showed a declining disposition.

### 3.2 Vitamin D deficiency hypothesis

The major natural source of vitamin D is dermal synthesis which is dependent on sun exposure, specifically, ultraviolet radiation. The decreased sunlight in winter leads to vitamin D deficiency. The vitamin D deficiency is a physiologically putative risk factor for TB infection and disease as it reduces the ability of macrophages to kill the intracellular *M*. *tuberculosis*. It has also been epidemiologically associated with tuberculosis disease [26]. According to the relevant studies, the peak month of vitamin D deficiency was midwinter (January in the regions of northern hemisphere and August in the southern hemisphere), followed by the spring peak of tuberculosis notification after consideration of symptoms onset and diagnosis delay [27].

If the vitamin D deficiency hypothesis is true, the latitude should be correlated to the TB seasonality. At the extreme latitude, there is insufficient UVB intensity in the autumn and winter months for adequate synthesis of vitamin D, and the SA of TB notification will be greater. These pieces of evidence were found from the studies in Australia [18] and India [10], which suggest that the seasonality of TB diagnoses was more pronounced in areas where UV exposure is reduced and vitamin D deficiency is prevalent. However, the amplitude of TB seasonality did not vary by latitude in the USA [14]. In our study, a different result showed greater TB seasonality in southern Xinjiang (34°N–43°N) than eastern and northern regions (43°N–48°N); however, it was not statistically significant. Nevertheless, this difference may be due to the relative latitudes of these locations: all latitudes of the continental USA are further from the equator (30°N–55°N) and those of Xinjiang China (34°N–48°N) are narrower than are latitudes of India (8°N–37°N) and Australia (10°S–44°S). On the other hand, exposure to sunlight is not only affected by the total hours of sunshine but individual factors also play a major role, such as occupation, culture, clothing and other personal habits. For example, the majority of the ethnic groups in Xinjiang are Muslim, and Muslim women are usually heavily covered for religious obligations, irrespective of the time of the year. The hours spent outdoors may vary with occupations. Some people may use sun protection in sunny weather and some others are not. These individual factors are not considered in the present analysis.

Our study suggested weak evidence for vitamin D deficiency hypothesis. Firstly, pleural TB cases had statistically significant greater SA than pulmonary TB cases. Secondly, relapsed cases had greater SA than new cases, though not statistically significant. Pleural TB and relapsed cases occur more commonly due to the reactivation than primary infection. Some researchers found that reduced winter sunlight and its potential effect on vitamin D levels decreased the immune competence, thereby increasing the endogenous reactivation of the latent infection [28]. Therefore, the greater seasonality of these cases supports the vitamin D deficiency hypothesis. However, the notification of pleural TB cases exhibit more reporting bias than pulmonary TB since the former is not mandatory to report, and hence, the credibility of this evidence should be considered.

### 3.3 The other explanations for TB seasonality

Other factors have been suggested as plausible explanations for TB seasonality; for example, co-infection with other seasonal pathogens, seasonal rhythm of nutrient intake, seasonal change in immune function, and healthcare seeking behaviors. Seasonality is observed in several infectious diseases and both viral and bacterial respiratory infections are more common in winter. Infection with other pathogens may accelerate the disease manifestation in patients with latent tuberculosis or increase the susceptibility to tuberculosis infection by decreasing the local immunity in the respiratory tract [29]. Malnutrition, a recognized risk factor of tuberculosis, is also common in winter because of variable availability of food and other nutrients. Many studies suggested that the competence of immune system vary by season with significant periodic changes in the amount of some peripheral blood leukocytes subsets and the immune cell function [30]. Therefore, the seasonal variability of TB notification with a peak in spring and/or summer is not surprising. The healthcare seeking behavior is also influenced by the seasons. Further delay in seeking healthcare would occur in winter season owing to the cold weather, resulting in a subsequent increase in TB notification in spring and summer.

## Limitations

Firstly, although the X-12-ARIMA approach used in this study was considered as the best seasonal adjustment program, there were some problems in its application in Xinjiang, China, because it cannot adjust the influence of local holidays such as Islamic festivals and spring festival. Thus, the development of a suitable seasonal adjustment program in the future is essential for Xinjiang, China. Secondly, ecological fallacy is the critical limitation of the present ecological study. Therefore, it can only provide some basis for the mechanism of TB seasonality rather than make causal inferences. Thirdly, the factors affecting the TB seasonality have not been well-studied owing to the limited information of patients in National Infectious Diseases Reporting System (NIDRS). Lastly, all the explanations of TB seasonality were only speculations. For example, young patients are more likely to be affected by recent exogenous infection; however, there were no objective indicators, such as analysis of clustered cases by genotyping methods, to determine those cases caused by recent infection. Therefore, further studies exploring the mechanism of TB seasonality by genotyping methods are essential.

## Conclusions

In conclusion, TB notification in Xinjiang, China, shows an apparent seasonal variation with a peak in the month of March and trough in the month of October. Although the precise cause responsible for the seasonal variation in TB notifications is unknown, several factors have been suggested as plausible explanations; for example, exposure to sunlight, indoor activity, co-infection with other seasonal pathogens, seasonal change in immune function, and healthcare seeking behaviors. Our results support the hypothesis that indoor crowding during the winter increases the risk of TB transmission, and the seasonality was mainly influenced by the recent exogenous infection rather than the endogenous reactivation in Xinjiang. The results of the present study provide evidence for the development of a control strategy to minimize the seasonal increase of TB cases. To the best of our knowledge, this is the first study using X-12-ARIMA seasonal adjustment method to investigate the seasonality of TB notification in Xinjiang, China. Data were collected over an extended period by the unit of monthly notification and also by different subgroups to provide evidence for potential determinants of TB seasonality.
